# Mixing a Grounded Theory Approach with a Randomized Controlled Trial Related to Intimate Partner Violence: What Challenges Arise for Mixed Methods Research?

**DOI:** 10.1155/2013/798213

**Published:** 2013-03-20

**Authors:** Cristina Catallo, Susan M. Jack, Donna Ciliska, Harriet L. MacMillan

**Affiliations:** ^1^Daphne Cockwell School of Nursing, Ryerson University, Toronto, ON, Canada M5B 2K3; ^2^School of Nursing, Faculty of Health Sciences, McMaster University, Hamilton, Canada L8S 4L8; ^3^Departments of Psychiatry and Behavioural Neurosciences and Pediatrics, Faculty of Health Sciences, McMaster University, Hamilton, Canada L8S 4L8

## Abstract

Little is known about how to systematically integrate complex qualitative studies within the context of randomized controlled trials. A two-phase sequential explanatory mixed methods study was conducted in Canada to understand how women decide to disclose intimate partner violence in emergency department settings. Mixing a RCT (with a subanalysis of data) with a grounded theory approach required methodological modifications to maintain the overall rigour of this mixed methods study. Modifications were made to the following areas of the grounded theory approach to support the overall integrity of the mixed methods study design: recruitment of participants, maximum variation and negative case sampling, data collection, and analysis methods. Recommendations for future studies include: (1) planning at the outset to incorporate a qualitative approach with a RCT and to determine logical points during the RCT to integrate the qualitative component and (2) consideration for the time needed to carry out a RCT and a grounded theory approach, especially to support recruitment, data collection, and analysis. Data mixing strategies should be considered during early stages of the study, so that appropriate measures can be developed and used in the RCT to support initial coding structures and data analysis needs of the grounded theory phase.

## 1. Introduction

Mixed methods research uses both quantitative and qualitative data to improve understanding of a research problem beyond what is possible with either approach alone [1]. Mixed methods studies are challenging for health researchers to plan and implement due to their design complexity and difficulties related to appropriate integration of data and results [2–4]. There is acknowledgement among health researchers about the value of mixed methods, but concerns regarding a lack of formal education and skills in using mixed methods [5]. While many investigators have received formal graduate training in either quantitative methods or qualitative methods, few had been exposed to the specifics of mixed methods designs and the process for mixing and integration of data [5]. There continues to be a gap in our understanding of how mixed methods can be used in health research, especially when combining two methods in a single study. While mixing two methods can offer a more comprehensive investigation of a research problem, there are unique challenges to integrating two methods.

### 1.1. Study Aim

Our goal is to describe the process of implementing a sequential explanatory mixed methods study involving a randomized, controlled trial (RCT) with a subanalysis of quantitative data and a qualitative grounded theory approach. Using an example study, we will explore how this mixed methods study was introduced after a large multisite RCT was in progress. The investigators felt that the mixed methods study would provide greater context and explanation for preliminary quantitative findings arising from the RCT. As a result of implementing a mixed methods study during the course of an RCT, methodological considerations were made in order to maintain the integrity of the trial and the mixed methods component—the quantitative subanalysis of emergency department site results from the RCT and grounded theory approach. The focus of this paper will be on describing the methodological modifications made to the grounded theory approach when used as an explanatory component for a RCT and then providing design and analytic considerations for the conduct of similar studies. The strengths and challenges of combining a RCT with a grounded theory approach in a mixed methods study will also be discussed.

### 1.2. Incorporating Qualitative Approaches with RCTs

The philosophical underpinnings of mixed methods have been extensively debated including whether or not qualitative methods should be mixed with quantitative methods [6–10]. These debates have focused on discussions about whether or not quantitative and qualitative research can be combined [6, 8, 10–13], the methodological challenges of combining quantitative and qualitative methods [9, 11–13], and the inconsistent integration of both quantitative and qualitative methods, most often with more emphasis on maintaining the integrity of quantitative methods [10, 11, 13–15]. Central to the philosophical debate is the concern about juxtaposing worldviews, such as combining positivist perspectives with that of social constructivist standpoints. The RCT is the gold standard in evaluating the effectiveness of an intervention on preidentified health outcomes [9, 10, 12–14, 16]. The RCT is grounded in the positivist worldview, where the goals are to (1) identify causal relationships leading to one empiric truth using deductive methods like hypothesis generation and prediction, (2) use control over the experimental environment to reduce the influence of potential extraneous variables, and (3) interpret the findings using statistical tests without consideration of the participant's interpretation [15, 17]. Qualitative methods, such as grounded theory, often follow the social constructivist worldview whereby the goal is to understand participant perspectives including the individual and multiple meanings allocated to phenomena, surroundings, behaviours, and social practices [15, 17]. Qualitative research emphasizes understanding individual perspectives as multiple truths and aims to aggregate the beliefs, social behavior, and processes that arise from participant perspectives and do not use the same practices or methods as with quantitative research [15, 17]. Because of these opposing philosophical stances, authors have described the debates about whether or not these two methods can be combined [5–17]. Teddlie and Tashakkori [18] stated that a focus on paradigmatic differences can halt the productivity of innovations in mixed methods research. Others have argued that a mixed methods design can help resolve the paradigm debate through its philosophical grounding in pragmatism which offers a utilitarian approach to research [18, 19]. Mixed methods permit a deeper and richer understanding of the phenomenon under study through an emphasis on plurality—of philosophical paradigms, theoretical assumptions, and methodological techniques [20]. Many authors recognize the multiplicity of paradigms which can compete and give rise to contradictions, but which is a normative component of mixed methods research [20]. This means that methods are combined, regardless of their individual philosophies, in order to meet the goals of the overall research project. Many investigators now value the need for mixed methods studies that incorporate qualitative and quantitative methods to better understand phenomena or to provide a richer explanation of results [6, 7, 11]. Qualitative research used within a RCT can explore the meaning that participants attribute to the intervention, which might serve to maximize its efficacy through greater understanding of context and patient experiences. Finally, using a qualitative approach to better understand the process of events and actions could add insight into the feasibility of the intervention [10]. Researchers have combined qualitative research with a RCT, so that the shortcomings of either method could be overcome while comprehensively addressing a research question, such as the effectiveness of a complex intervention [13]. For this study, we acknowledge this debate but have adopted a pragmatic approach [17–19] to the use of qualitative and quantitative methods together for study.

Lewin et al. [11] in their systematic review of RCTs of complex interventions randomly retrieved 100 out of 492 eligible studies to explore the use of qualitative methods to understand RCT results. In the 100 RCTs, only 30 incorporated qualitative studies to explain their results. Among the 30 RCTs that did involve formal qualitative approaches, the authors found that there was limited rationale provided for mixing methods, little discussion of the integration of methods, and few studies presented the contributions of both quantitative and qualitative methods to overall study interpretations [11]. The following methodological flaws were identified with the qualitative studies: limited explanation of the design/approach, lack of discussion regarding recruitment and sampling methods, failure to describe modifications to data collection methods, and poor description of qualitative data analysis and thematic development [11]. These methodological flaws in the qualitative studies accompanying intervention trials were attributed to a lack of expertise in qualitative methods and resource constraints to carry out both a RCT and a qualitative study [11].

Kinn and Curzio [13] conducted a systematic review that assessed the incorporation of quantitative and qualitative methods in research articles. The authors searched MEDLINE, CINAHL, and PsycInfo bibliographic databases from 1982 to 2000. Fourteen studies from 2,382 citations retrieved and reviewed for relevance were selected for appraisal. Those appraised were mixed methods studies, some of which included a RCT. Findings indicated that qualitative approaches were used for different purposes, ranging from preliminary exploratory work to validation of quantitative findings [13]. Similar to Lewin et al. [11], Kinn and Curzio [13] found that the qualitative component of the mixed methods studies were poorly described such as the qualitative design selected, sampling procedures, coding and analysis techniques, and the methods for the integration of both quantitative and qualitative findings.

Other authors have confirmed these systematic review results and emphasized the need for greater cohesion of methods when incorporating qualitative approaches with a RCT [14, 21]. Combining methods in a transparent manner can help overcome methodological weaknesses in both approaches and provide a comprehensive examination of the research question under study [16]. The most advantageous use of qualitative approaches with the randomized trial occurs when both hold equal importance and weighting and are not considered subservient to one another [14, 21].

## 2. Materials and Methods

### 2.1. Example of a Mixed Methods Study

**Figure 1:**
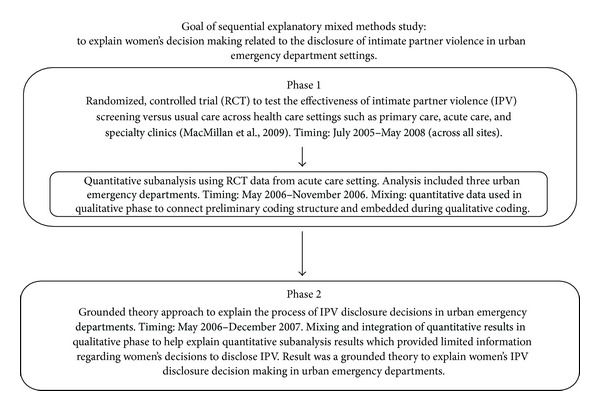
Description of sequential eeplanatory mixed methods study to explain IPV disclosure in emergency departments.

Sequential explanatory mixed methods designs aim to answer one type of question by collecting and analyzing two types of data (qualitative and quantitative) and drawing inferences using both data types usually during the interpretation stage of the study in two distinct phases [1, 22]. With the sequential design one type of method (quantitative or qualitative) is timed to be completed first from data collection to analysis (e.g., Phase 1) before beginning the second method (e.g., Phase 2). The results from the first phase help to inform the second phase of data collection and analysis involving the second method [1]. The primary purpose of the sequential explanatory design is to explain and expand upon findings identified during the first phase of the study [1, 23]. In our example, the purpose for this sequential mixed methods design was to enhance initial quantitative results by using followup qualitative methods. This mixed methods study was initiated a few months after a multisite RCT was underway. The RCT examined the effectiveness of routine screening for intimate partner violence (IPV) in health care settings compared with usual care in reducing violence and improving life quality; its methods are reported elsewhere [24]. Initial RCT results from the emergency department sites indicated that some participants disclosed IPV to the emergency department health care provider, yet the specific nature of the decisions leading to this disclosure, including the processes that abused women used to disclose, could not be described. As a result, a mixed methods study was identified as the most ideal method for this study, as it could provide a more comprehensive analysis of the quantitative data from the RCT emergency department sites; a qualitative component could expand on these results to better understand abused women's decision making regarding disclosure of violence in urban emergency department settings in ON, Canada. The first phase of this mixed methods study was quantitative and involved a subanalysis of RCT data from three emergency department sites including: demographic information; two screening instruments to assess women's report of IPV (1) the Composite Abuse Scale (CAS) [25] and (2) the Woman Abuse Scale Tool (WAST) [26]; and a verifying report from the health care provider that the subject disclosed IPV. The quantitative subanalysis results helped to describe rates of IPV and the proportion of participants that disclosed violence to an emergency department health care provider. However, these results did not explain why those women who scored positive for IPV decided not to disclose during their visit to the emergency department. The quantitative results also did not describe the problems that women perceived with disclosure or the processes used by women who did disclose violence in the emergency department setting. After review of these quantitative results, grounded theory was considered the best qualitative method to explain these findings due to its ability to both describe and explain a system of behaviour and seek, as an end result, a substantive midrange theory grounded in the data [27]. Grounded theory aims to build a midrange, substantive theory that portrays a central problem, its characteristics and contributing factors, the process and strategies used to resolve the problem, and the consequences if the problem was not resolved [28]. In this example study, we sought to understand what women saw as the central problem related to disclosure of IPV to an emergency department health care provider and the strategies and processes that they used to resolve the problem related to disclosure. Traditional grounded theory was selected to guide data collection, organization and data analysis [28–30] (Phase 2) (Figure 1).

### 2.2. Phase 1: Subanalysis of RCT Data

#### 2.2.1. Sampling and Data Collection

Sampling for mixed methods studies follows the general rules governing both quantitative and qualitative methods and involves selecting participants using both probability and purposive sampling techniques [31]. According to Collins et al. [32], determining an appropriate sampling scheme is pivotal to the preservation of rigour and the overall integration of results for a mixed methods study. MacMillan et al. [24] provided details of the overall RCT randomization and recruitment process. For this quantitative subanalysis phase of the mixed methods study, participants in the RCT were selected from May to November 2006, across three emergency department settings, and those who met the following eligibility criteria were recruited:

- 18 years or older and previously enrolled in the RCT at one of the three recruitment emergency departments;
- recruited into the RCT and scored positive for IPV using the WAST and the CAS (details of instruments in MacMillan et al. [24]);
- able to disclose IPV to a health care provider during their emergency department visit;
- healthy enough to participate in a 60–90 minute interview;
- able to speak, read, and write in English.

In addition to the WAST and CAS, an additional instrument called the clinical interactions instrument was completed by participants who disclosed IPV during their visit to the emergency department. This instrument involved participants selecting the type of intervention that they were offered by the health care provider after disclosure of IPV.

#### 2.2.2. Analysis

Descriptive statistics was used to analyze the quantitative results using SPSS version 17. Data were analyzed to determine the rates of IPV exposure using the screening instruments from the RCT and the rates of disclosure to an emergency department health care provider.

### 2.3. Phase 2: Grounded Theory Study

#### 2.3.1. Sampling and Data Collection

After the analysis of quantitative data was completed for Phase 1, the second phase involving a qualitative method began. The quantitative results were used to help inform the grounded theory study, and all participants were recruited from the RCT subanalysis sample that met eligibility criteria (*n* = 174) and interviewed from May 2006 to December 2007. For the grounded theory component of the mixed methods study, an estimated goal to sample 20–27 women was identified in keeping with other grounded theory studies involving participants who were either deemed vulnerable or who attended acute care settings [33, 34]. Theoretical sampling and saturation guided sampling and data collection for the grounded theory phase. According to Schwandt [35], theoretical sampling includes collecting data on activities and events as dictated by the evolving theory. Continuous comparison is an essential feature of grounded theory where comparisons are made between the developing theory and the raw data until no new findings or views emerge regarding a concept or category—a key feature of saturation [35]. Constant comparison is a key element of grounded theory where comparisons are made between the developing theory and the raw data until no new findings or views emerge regarding a concept or category [36]. In order to begin the process of developing codes and categories to drive initial theoretical sampling, four participants were recruited from the quantitative subanalysis. Criteria were created to identify variation among participants and build codes that would drive future interviews prior to entering the theoretical sampling phase of the study [27]. Once a preliminary set of codes and interview guide were developed, eligibility for inclusion continued to focus on theoretical sampling, which is an integral part of the analytic process for grounded theory. Data were simultaneously collected, coded, and analyzed using activities to saturate the evolving theory and increase its level of abstraction [29, 36]. During theoretical sampling, our goal was to obtain conceptual density and a theory grounded in the data, despite a bounded sample of participants from Phase 1 of the mixed methods study. As a result, we used the constant comparison of findings was undertaken based on the incidents, events, or situations related to the disclosure of IPV in urban emergency departments [30, 37]. We called these “IPV disclosure events”, and this “unit of analysis” approach allowed us to maximize potential for theoretical density using constant comparison across IPV disclosure events [29]. This meant that we analyzed over 100 intimate partner violence disclosure histories and events (units) involving emergency department health care providers, health care providers in other settings, and other professionals [29] in order to identify features and components for developing concepts and categories [36]. The unit of analysis did not represent actual participants but rather events, incidents, or examples. The unit of analysis approach was also used during various stages of coding.

In addition to recruiting participants based on “IPV disclosure events”, as a means of achieving theoretical saturation, we also recruited participants who scored positive on the WAST and negative on the CAS measures used in Phase 1 of the mixed methods study or those who did not disclose IPV to a health care provider during their visit to the emergency department. Once the core phenomenon was defined and relationship linkages developed, two other sampling methods used to assess the developed categories and to raise the description of the theory to a higher level of abstraction [36]. Maximum variation sampling, sometimes referred to as extreme case sampling, involves interviewing participants who hold different perspectives, sometimes at “opposite ends of the spectrum” in order to obtain a full understanding of the phenomenon under study [1, 31]. Typically, this involves locating and recruiting new participants into the grounded theory study [36]. Negative case sampling was also used as an approach to help assess the developing grounded theory. The goal of negative case sampling is to test emerging theoretical hypotheses in order to achieve a higher level of theoretical abstraction beyond a description of relationships between concepts [36]. This type of sampling is typically performed after the development of the grounded theory in order to identify new participants whose experiences refute or deviate from the developing theory [31, 36]. Deviant cases offer opportunity for comparison across other categories and are often selected outside of the sample used to generate the grounded theory [36].

Data were collected in face-to-face, in-depth, and semistructured interviews lasting from 60 to 90 minutes from May 2006 to December 2007. All interviews were digitally recorded and conducted in a private location determined with the participant; all participants were provided with a $25 honorarium per interview. Interview guides were used at each interview and were modified during analysis to capture the developing categories and themes and to enhance saturation.

#### 2.3.2. Analysis

Three levels of coding were used for the grounded theory phase including “in vivo,” substantive, and theoretical coding. Throughout the coding process, the constant comparison method [28] was used to review data for fit and relevance, to evaluate codes with one another to generate categories, and to identify relationships between categories for the ultimate generation of a theory grounded in the data [30]. During “in vivo” coding, codes were labelled, separated, and compiled to capture the main idea of the participant (Level 1) [36]. These codes were then grouped together to create clusters or families which were then classified under a label as “concepts.” These concepts were then organized together to form categories (Level 2) [36, 37]. Constant comparison identified relationships among codes and categories (Level 3), properties and dimensions of the categories, and conceptual linkages within the data [30]. With grounded theory, the goal is to identify a core phenomenon and basic social psychological problem (BSP) [28]. Once these were identified from the data, selective coding strategies were used to build categories and extend theoretical abstraction.

## 3. Results

### 3.1. Phase 1: Subanalysis of RCT Data

The sample consisted of 1182 participants across three RCT emergency department sites.  Table 1 outlines the main demographic characteristics of participants. On average, participants were 25–34 years old, in common-law relationships, had an average of 13.9 years of education, worked full or part time outside of the home, and had an annual household income in the $40,000–$62,000 range. Analysis involving the WAST and the CAS instruments identified that 14.7% (*n* = 174) women reported a true positive score for IPV exposure. For the remaining participants, 73.6% (*n* = 870) indicated a true negative score for IPV, 10% (*n* = 118) a false positive score, and 1.7% (*n* = 20) a false negative score as seen in Table 2. The rate of mixed IPV exposure status (i.e., false positive or false negative score) remained consistent across both groups. Among the 174 participants with a true positive score for IPV exposure, only 1.9% (*n* = 22) indicated that they had disclosed violence to an emergency department health care provider during their visit. Among the women who disclosed IPV in the emergency department, the following interventions were reported most frequently as being offered to them: information regarding options to obtain help (*n* = 15), acknowledgement of IPV disclosure (*n* = 13), verbal information about IPV (*n* = 13), and assessment of immediate safety (*n* = 13). Less frequently reported interventions offered to women were a discussion about the relationship situation and inquiry about the woman's need for help (*n* = 8), a validation of the woman's feelings (*n* = 6), and decision making about the type of health to obtain (*n* = 1).

### 3.2. Phase 2: Grounded Theory Study

**Figure 2:**
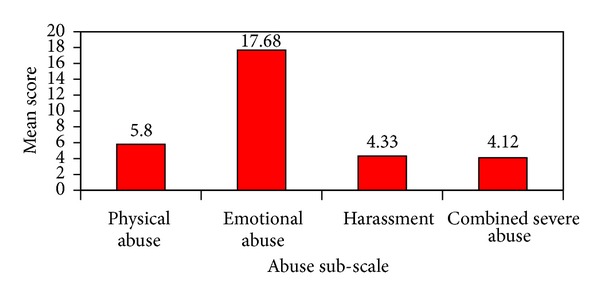
Mean scores for composite abuse scale subscales.

The grounded theory phase began after the analysis of quantitative data was completed, and recruitment involved 20 participants from the quantitative subanalysis phase. Early in this phase, data from one participant had to be removed when her responses raised concerns about the validity of the information, leaving a final sample of 19 participants. Fifty interviews were completed across the 19 participants. Seven participants each completed four interviews. The remaining 12 participants completed from one to three interviews. The data from 50 interviews identified over 100 IPV disclosure events involving emergency department nurses and health care professionals, social service professionals, police, family members, and friends. Using the WAST, 16 participants scored positive (≥4), and three scored negative (<4) IPV exposure (the full range of scores from 0 to 16 points). The mean score value across all participants was 8.03. Using the CAS, 18 participants scored positive (≥7) for IPV with a mean score value of 31.9 (the full range of scores from 0 to 150 points). Using the subscales of the CAS, 10 participants reported combined severe abuse (mean = 4.12); 13 reported physical abuse (mean = 5.80); 18 reported emotional abuse (mean = 17.68); and 11 reported harassment (mean = 4.33) (Figure 2). For 15 participants the prevalence of emotional abuse was higher than other forms of abuse. “Being found out” was the basic social psychological problem among women exposed to IPV, as they feared that the health care provider would learn about the abuse during the provision of care at the emergency department. Women undertook a basic social psychological process of “minimizing the risk of intrusion” from health care providers when deciding whether to disclose IPV. The process included three phases that women used to minimize their risk of intrusion from health care providers including: deciding how and when to seek emergency care, evaluating how well they trusted emergency department health care providers, and establishing a personal readiness to disclose. Consequences of this process were a self-initiated disclosure of IPV in the emergency department, perceived forced disclosure of IPV when women felt invaded, and a failure to disclose IPV because women were not ready to disclose or could not trust the health care provider. More information and detail regarding the grounded theory results can be found in Catallo et al. [29].

### 3.3. Integration of Quantitative and Qualitative Data

Mixing of the quantitative and qualitative data occurred at various points and two key mixing techniques were used in this study, embedding and connecting data. Mixing began with connecting data which involves the analysis of one type leading to the need for another type of data [1]. Connecting the quantitative subanalysis data to the developing qualitative data through review of the RCT subanalysis results helped to develop the first interview guide and initial coding structure. The data obtained from the WAST, CAS, and clinical interactions instrument were used to inform participant selection, initial research questions, and the development of original interview guide. For example, the WAST and CAS were used to identify IPV exposure and recruit potential participants from each of the following groups: true positive score, true negative score, and mixed scores (i.e., false positive and false negative). Quantitative results from all of the instruments were used to develop initial qualitative interview questions and an interview guide. For example, results from the clinical interactions instrument indicated that a low percentage of women who were exposed to IPV disclosed in the emergency department (i.e., 1.9%), and interview questions were developed to understand why women either chose to disclose IPV or not and the nature of the interventions offered to women. Example questions from the interview guide that were developed through the integration of quantitative subanalysis data can be found in Table 3. In addition to this data-connecting strategy, quantitative subanalysis results were embedded to support the analysis and interpretation of the qualitative data. Embedding data can involve mixing at the design level where one form of data is embedded in another form and can occur sequentially [1]. Embedding quantitative data during qualitative analysis helped to support theoretical coding and this drove decisions about continued sampling of participants. For example quantitative results were embedded into the grounded theory approach by analyzing categories of IPV exposure as defined by the CAS [25]. These data were then used during theoretical coding to identify potential relationships between emerging categories and to create constructs in order to generate meaning for the developing theory [38]. The quantitative subanalysis results were embedded in the qualitative analysis in two ways: (1) to compare participants according to low, medium, and high scores for violence exposure and (2) to compare participants according to different types of IPV using the measures' subscales. Participants were then grouped according to their scores:

1. low (0–4 points WAST, 0–29 points CAS),
2. moderate (5–9 points WAST, 30–59 points CAS),
3. high (10–16 points WAST, 60–150 CAS).

Grouping participants according to severity of IPV exposure aided in qualitative analysis and interpretation of the data when identifying codes and categories. Participants with high scores were most concerned with being judged by health care providers for remaining in an abusive relationship. We used this result to focus our qualitative analysis on this subgroup, so that we could assess unique features to enhance the understanding of the properties of the developing categories, especially those central to grounded theory such as the core phenomenon and basic social problem. After identification of the basic social problem, participants were compared across low, moderate, and high levels of violence severity to verify existing categories and relationships or identify any new features or conditions not seen previously. In the second mixing strategy, CAS results were used to organize qualitative participants according to the type of abuse including combined, severe abuse, physical abuse, emotional abuse, and harassment. This was completed to identify or confirm emerging or developing relationships. Participants with high CAS scores for emotional abuse had more difficulty identifying their relationship as abusive and seeking care for violence, unless it was for an immediate physical injury. Participants who scored high for more than one type of abuse, like severe combined and emotional abuse, avoided health care as this group perceived the risks of exposure, and were too great. Embedding the quantitative data during qualitative analysis permitted exploration of these relationships and categories to provide additional explanation.

The process of maximum variation sampling required modification to conduct the grounded theory phase. Typically, when this type of sampling is used in grounded theory, new participants outside of the study are sought to test the full spectrum of a category's properties and dimensions, including that of the core variable phenomenon as well as the developing relationship between categories [30]. Our purpose was to understand violence disclosure from the perspective of the RCT participants, so we did not recruit participants from outside of the trial. We addressed maximum variation sampling by examining variants of intimate partner violence IPV disclosure events at opposing spectrums. This was used when identifying the properties and dimensions of a concept, testing developing relationships and theoretical hypotheses. This resulted in recruiting participants with mixed scores and negative scores on the intimate partner violence IPV screening measures. For example, maximum variation sampling was used in this study to identify the dimensions of the category “building trust with a health a care provider.” Disclosure events describing a participant's ability to trust health care providers with extreme ease were compared against disclosure events in which participants described extreme difficulty. Studying these units of analysis allowed for further development of the category by identifying subcategories and relationships. One example was to compare the units of analysis for participants who could immediately trust health care providers (implicit trust), participants who trusted clinicians after a demonstration of trustworthiness (distrust), and those participants having extreme difficulty trusting health care providers (mistrust). When more information was required, the unit of analysis was linked back to the original participant, and that participant was sought for further interviews.

For this mixed methods study, we modified the negative case sampling method used in grounded theory. The goal of negative case sampling is to test emerging theoretical hypotheses in order to achieve a higher level of theoretical abstraction beyond a description of relationships between concepts [36]. This type of sampling is typically performed after the development of the grounded theory in order to identify new participants whose experiences refute or deviate from the developing theory [31, 36]. Deviant cases offer opportunity for comparison across other categories and are often selected outside of the sample used to generate the grounded theory [36]. Because we recruited participants only from the RCT subanalysis (Phase 1), we had to modify negative case sampling for use with the unit of analysis approach. Instead of sampling for negative cases, disconfirming evidence related to IPV disclosure events was sought from the data. Disclosure events were reviewed when theoretical hypotheses were generated in order to determine potential contradictory relationships that could test or challenge the developing theoretical hypotheses. This is referred to as “negative case analysis” and was deliberately completed during the different stages of coding and analysis, not just after a full theory was developed [39]. For example, we explored IPV disclosure events where participants perceived a positive outcome after disclosure such as obtaining a referral, support, or other interventions to support their safety. We then looked for disconfirming IPV disclosure events, such as an IPV disclosure where participants perceived a negative outcome after disclosure such as feeling intruded upon by the health care provider, feeling forced to involve police services, or being investigated by child protective services. During our negative case analysis we examined these IPV disclosure events that disconfirmed what we were seeing in the data. In this example, we sought to recruit or offer additional interviews to participants who had perceived negative disclosure outcomes, so that we could better understand disconfirming cases and add conceptual density to our developing theory. Another example of negative case analysis occurred during the analysis of the basic social psychological process used by abused women to minimize the intrusion that they perceived in the emergency department. The first phase of these process involved decisions about whether or not to seek care at the emergency department when IPV might be “found out” by the health care provider. Through negative case analysis we sampled IPV disclosure events where women avoided treatment at the emergency department at all costs, even at risk of death. As a result, we theoretically sampled events where participants actively engaged in avoiding health care and the meaning of these actions. In addition to sampling for disclosure events, we sought to recruit participants who actively avoided the emergency department for fear of IPV being found out by the health care provider. Because many diverse units of analysis were available for review, negative case analysis could be conducted. When more information was required, the unit of analysis was linked back to the participant who underwent additional interviews to provide greater insight. However, this approach would not have been possible had there been a small sample of disclosure events or if the events from which to draw on had been very similar in nature. While negative case sampling—which in its truest form tests the full theory after completion among new participants—could not be performed, the principles underlying this approach were maintained. Negative case analysis using disclosure events as the unit of analysis provided the researcher with an opportunity to evaluate contradictory developments, as the grounded theory was developed and refined.

### 3.4. Timing of Data Collection

Unique decisions about data collection were made in this mixed methods study. At the time of ethics approval, initial recruitment for the RCT had been completed or was near completion for certain settings (e.g., primary care and specialty clinics). Recruitment among the acute care settings for the RCT was still at an early stage. This was fortuitous for the mixed methods study, as it allowed the most time for ongoing analysis and data collection for both the quantitative subanalysis and the qualitative explanatory phase. There were occasions when participants were sought for the grounded theory phase but were no longer being followed by the RCT. When this sampling need was identified, the acute care recruitment was completed for the RCT. This created challenges for the grounded theory phase, as participants who had completed the trial were no longer interested in participating in an additional study, extending the time of recruitment.

## 4. Discussion

This study provides new insights into the use of a grounded theory approach within the context of an RCT. This study provides a unique description of how a grounded theory study was used with RCT data to provide greater explanation for quantitative results. We address a current gap in nursing research by outlining explicit strategies for mixing quantitative and qualitative methods and describing how these were integrated to inform the overall study, typically an area that is overlooked when using a qualitative approach with a RCT. Mixed methods research can make an important contribution to nursing where many clinical issues require a comprehensive and holistic approach which requires data from various perspectives [40]. A unique contribution to nursing research is this study's use of grounded theory as an equally weighted approach alongside the RCT to improve the depth and richness of results when examining a complex intervention. More typically we identified sequential explanatory mixed methods studies with heavier weighting in the quantitative component [10, 13, 14, 16, 17, 21]. Other research areas in nursing can benefit from this type of mixed methods design such as the evaluation of nursing interventions, exploration of patient centred care, in-depth exploration of complex phenomena, and instrument development and testing. Our study's weighting between quantitative and qualitative phases enabled a more thorough understand of a complex phenomenon like IPV disclosure in emergency departments that would not have occurred using a RCT alone. More common is the use of qualitative types of data collection, such as open-ended questions, to help explore RCT results or to understand implementation issues specific to the RCT [7, 11]. We identified that it is possible to integrate a qualitative approach with an RCT if modifications are made, in this case to the grounded theory sampling strategies of maximum variation and negative case sampling. Using the “unit of analysis” approach during the grounded theory phase enabled us to maintain the principles of theoretical sampling in accordance with traditional grounded theory methods and offer pragmatic adaptations that could be used to strengthen the sampling when using grounded theory in a mixed methods study. Despite adaptations to the grounded theory, we still maintained methodological rigour which made it possible to combine with a RCT. Because our quantitative subanalysis originated from strict principles governing a RCT, namely, randomization with predetermined inclusion and exclusion criteria, participants had an equal and unbiased opportunity to be randomly allocated to either the experimental group (i.e., routine screening) or the control group (i.e., usual care). This added strength to the recruitment strategy for the study, as random selection and allocation are important ways of reducing error and selection bias [41].

It is important to outline the key limitations associated with incorporating a grounded theory approach with a RCT. The most significant limitation of this study was beginning this mixed methods study after the overall RCT was underway. Creswell and Plano Clark [1] recommend deliberately planning a mixed methods study as study in and of itself. The RCT was highly complex and involved multiple health researchers, so it was difficult to estimate the types of mixed methods research questions that would arise. It was challenging to predict the need for a mixed methods study at the outset of the RCT. The pragmatic approach, in keeping with the philosophical principles of mixed methods design, was to incorporate a mixed methods study into this developing research program. This enabled the identification of unique research opportunities that would have otherwise been missed.

An additional challenge that arose in incorporating a qualitative approach with a RCT was the large amount of time needed to complete both phases of the study. During the grounded theory phase, it was necessary to recruit participants who had previously completed the RCT. Because these participants were no longer involved, it was more difficult to reengage their interest for the qualitative component. As a result, it is uncertain what the overall impact was for having fewer participants from the group who were no longer followed by the RCT on the developed grounded theory.

## 5. Future Recommendations

The following recommendations should be considered when incorporating a qualitative approach such as grounded theory with a RCT as part of a mixed methods study.

### 5.1. Design Mixed Methods Studies during Proposal Development

For this study, we adopted a pragmatic approach to design and implement a mixed methods study after the commencement of a multisetting RCT. The mixed methods design was chosen as a result of secondary research questions that arose from preliminary trial data. Ideally mixed methods studies should be planned for during proposal development and include specific research questions driving the mixed methods study. Future research teams need to create environments where the norm is to integrate qualitative results into trials examining complex interventions. There is a growing consensus that combining quantitative and qualitative research methods can provide a more comprehensive understanding of complex interventions, especially to address the varied information needs of policymakers, decision makers, and stakeholders [42–44]. In addition, the use of both types of data can contribute to the evaluation of the intervention including a broader understanding of its context and justification for its use in patient populations [6, 42, 44]. In the early stages of a mixed methods study involving a randomized trial, early planning to include a qualitative component can help examine trial implementation such as challenges to study recruitment, attrition rates of participants, and patient preferences regarding the intervention [43]. Planning for a mixed methods design at the outset of a study will enable consideration of the design needs including the implementation of the quantitative and qualitative components and opportunities for mixing the two data sets.

### 5.2. Planning for Potential Data Mixing Opportunities

While this study involved both the embedding and connection of quantitative and qualitative data as mixing strategies, future mixed methods studies should seek and incorporate a greater number of opportunities for data mixing using both RCT results and qualitative results [5, 42, 43]. At minimum, data mixing needs to support the explanatory phase of the sequential mixed methods study, in our case the grounded theory phase. While we used data mixing to support the earlier stages of the grounded theory phase (e.g., initial recruitment, development of an early interview guide, coding, and analysis) an improvement would be to plan for multiple data mixing opportunities such as the early and later stages of the explanatory phase to aid in recruitment, data collection, and analysis. For our study, in order to use more of the quantitative data in the later stages of the theory development, we would have needed additional quantitative measures beyond what was used in our quantitative subanalysis phase. For example, an instrument that explored participant decision making regarding IPV disclosure in the emergency department setting could have produced results that would aid in conceptual density of the developing theory on minimizing intrusion from health care providers for women seeking to disclose IPV. This suggests that those undertaking this type of study in the future need to incorporate multiple types of measures, so that the data can be better used at different stages of the qualitative phase.

### 5.3. Timing of Quantitative and Qualitative Phases

This study found that the timing of both the quantitative phase and the qualitative phase was crucial in terms of recruitment, reducing attrition, and analysis. When incorporating a grounded theory approach with a RCT, adequate time should be allotted, so that the initial interviews for the grounded theory approach can be completed during the data collection phase of the trial. Because grounded theory requires simultaneous data collection, coding, and analysis [30], it may take longer time periods to identify the participant characteristics required for ongoing sampling. For this study, we required 18 months and up to four interviews per participant to carry out theoretical sampling and build conceptual density of the developing theory. Because the subanalysis of data had been completed earlier, it was more challenging to maintain contact with participants for the length of time needed in the grounded theory phase of the study. Those planning such a study in the future need to consider additional time needed such as extending RCT recruitment time or maintaining participant engagement during periods when they were not being interviewed in order to reduce attrition.

### 5.4. Provision of Appropriate Expertise to Support Phases

Implementing a grounded theory with a RCT requires human resources support, especially when studying a highly vulnerable and transient sector of the population. Proposal planning for a mixed methods study should take into consideration the human resources need to carry out both the quantitative and qualitative components of the study. When using grounded theory with an RCT, it is necessary to have a research team with expertise in both trial and qualitative methodologies. A team with expertise in both methods will help to identify challenges to study implementation and effective strategies to address these while maintaining the overall rigour of both methods.

In conclusion we described a mixed methods study, which involved the mixing and interpretation of a quantitative and qualitative data sets in order to explain how women decided to disclose IPV in emergency departments. We have strived to address some of the methodological gaps in the literature by providing a rationale for the mixing of methods, describing how qualitative methods were modified in order to maintain the rigour of the overall mixed methods study and outlining the methods used for qualitative data analysis and thematic development.

Using grounded theory enabled us to understand results from an RCT quantitative subanalysis and provided a comprehensive examination of the problems that women identify with IPV disclosure and the strategies that they use to address these problems. In order to incorporate a grounded theory approach with a RCT, key modifications to sampling and data analysis were required. Incorporating a qualitative approach with a RCT can enhance the study's overall findings and provide better explanations regarding incongruent findings. Key recommendations for use of a qualitative approach with a randomized trial include attention to timing, mixing, and the incorporation of appropriate expertise to carry out both approaches.

## Figures and Tables

**Table 1:** Quantitative subanalysis results: demographic summary across screen and nonscreen groups.

	Frequency (n)	Percentage	Mean
Age in years for total sample (*N* = 1182)			25–34 years
18–24 years	259	21.9	
25–34 years	366	31.0	
35–44 years	262	22.2	
45–54 years	193	16.3	
55–64 years	90	7.6	
Unknown/not reported	12	1.0	
Marital status for total sample (*N* = 1173)			Common law
Single, never married	342	29.1	
Married	66	5.6	
Common law	496	42.2	
Separated	59	5.0	
Divorced	203	17.3	
Widowed	8	0.7	
Missing	8	0.7	
Pregnancy status for total sample (*N* = 1182)			No
Yes	46	3.9	
No	1086	91.9	
Donot know	49	4.1	
Missing	1	0.1	
Number of children at home for total (*N* = 1182)			1.45
No children	533	45.1	
1 or more children	649	54.9	
Years of education for total (*N* = 1182)			13.92
Less than 14 years	558	47.2	
Greater than 14 years	624	52.8	
Main activity for total (*N* = 1182)			Work full or part time outside of the home
Work full or part time outside of the home	778	65.8	
Homemaker, student, unemployed, and disabled	404	34.2	
Main source of income for total (*N* = 1182)			Wages or salary
Wages or salary	430	36.4	
Partner's income, alimony or child support, and social assistance	714	60.4	
Missing	38	3.2	
Household income for total (*N* = 1182)			$40,000–$62,000
Less than $24,000	279	23.6	
$24,000–$39,999	287	24.3	
$40,000–$62,999	246	20.8	
$63,000–$89,999	196	16.6	
$90,000 and over	174	14.7	

**Table 2:** Quantitative subanalysis results: summary of intimate partner violence exposure status across screen and nonscreen groups.

Participants number (percent) *N* = 1182	Woman abuse screening tool (WAST) score	Composite abuse scale (CAS) score	Overall intimate partner violence exposure status
174 (14.7%)	Positive	Positive	True positive score
118 (10.0%)	Positive	Negative	False positive score
20 (1.7%)	Negative	Positive	False negative score
870 (73.6%)	Negative	Negative	True negative score

**Table 3:** Mixing of quantitative and qualitative data: use of quantitative data to develop initial qualitative interview guide.

Example interview guide questions	
(i) How would you describe your experience with the doctor or the nurse when you were in the emergency department? Probe: for nature of the interaction, what promotes comfort, what are barriers to comfort, and how the participant defines care and quality of care.	
(ii) What is your opinion about discussing intimate partner violence with an emergency department doctor or nurse?	
(iii) What are some of the benefits/difficulties in talking with a doctor/nurse about violence? Other questions relate to identifying: barriers and facilitators, how participant would go about talking about IPV with a doctor or nurse, what issues related to IPV would/would not be discussed with a doctor/nurse.	
(iv) You mentioned that you are open and willing to talk about violence in an emergency department (ED) any time. Can you think of any reasons why you would avoid talking about abuse in an ED? What steps do you take to avoid talking about abuse?	
(v) Can you think of the steps that you take before getting ready to talk to a doctor/nurse about violence in your relationship? Are there different steps you would take when talking to a nurse?	
